# Natural Product Chemistry of Gorgonian Corals of the Family Plexauridae Distributed in the Indo-Pacific Ocean 

**DOI:** 10.3390/md10112415

**Published:** 2012-11-01

**Authors:** Li-Hsueh Wang, Jyh-Horng Sheu, Shih-Yao Kao, Jui-Hsin Su, Yung-Husan Chen, Yu-Hsin Chen, Yin-Di Su, Yu-Chia Chang, Lee-Shing Fang, Wei-Hsien Wang, Yang-Chang Wu, Ping-Jyun Sung

**Affiliations:** 1 National Museum of Marine Biology and Aquarium, Pingtung 944, Taiwan; Email: wanglh@nmmba.gov.tw (L.-H.W.); sweetcloud0906@gmail.com (S.-Y.K.); x2219@nmmba.gov.tw (J.-H.S.); tony_chen72001@yahoo.com.tw (Y.-H.C.); kb5634@yahoo.com.tw (Y.-H.C.); gobetter04@yahoo.com.tw (Y.-D.S.); jay0404@gmail.com (Y.-C.C.); whw@nmmba.gov.tw (W.-H.W.); 2 Graduate Institute of Marine Biotechnology and Department of Life Science and Institute of Biotechnology, National Dong Hwa University, Pingtung 944, Taiwan; 3 Department of Marine Biotechnology and Resources and Division of Marine Biotechnology, Asia-Pacific Ocean Research Center, National Sun Yat-sen University, Kaohsiung 804, Taiwan; Email: sheu@mail.nsysu.edu.tw; 4 Doctoral Degree Program in Marine Biotechnology, National Sun Yat-sen University and Academia Sinica, Kaohsiung 804, Taiwan; 5 Department of Sport, Health and Leisure, Cheng Shiu University, Kaohsiung 833, Taiwan; Email: lsfang@csu.edu.tw; 6 School of Chinese Medicine, College of Chinese Medicine, China Medical University, Taichung 404, Taiwan; 7 Natural Medicinal Products Research Center, China Medical University Hospital, Taichung 404, Taiwan; 8 Center for Molecular Medicine, China Medical University Hospital, Taichung 404, Taiwan

**Keywords:** Plexauridae, gorgonian, *Astrogorgia*, *Bebryce*, *Echinomuricea*, *Euplexaura*, *Menella*

## Abstract

The structures, names, bioactivities and references of 105 natural products obtained from gorgonian corals belonging to the family Plexauridae with an Indo-Pacific distribution are described in this review. All compounds mentioned in this review were obtained from gorgonian corals belonging to the genera *Astrogorgia*, *Bebryce*, *Echinomuricea*, *Euplexaura* and *Menella*.

## 1. Introduction

Over the past thirty-four years, 105 natural products have been reported from gorgonian corals belonging to the genera *Astrogorgia*, *Bebryce*, *Echinomuricea*, *Euplexaura *and *Menella* with an Indo-Pacific distribution, all belonging to the family Plexauridae (Cnidaria: Anthozoa: Gorgonacea) [1]. This review summarizes the structures, names, bioactivities and references of all compounds in tabular form. 

## 2. Natural Products from Gorgonian Corals Belonging to the Family Plexauridae

### 2.1. Genus *Astrogorgia*

#### *Astrogorgia* sp. 

A novel 9,10-secosterol, astrogorgiadiol (**1**), and a new eunicellin-based diterpenoid, astrogorgin (**2**), along with a known eunicellin, ophirin (**3**), were isolated from the gorgonian *Astrogorgia* sp., collected at Okino-shima Island off Shikoku, Japan [2] (Table 1). The structures of new metabolites **1** and **2** were established by spectroscopic methods and by comparison of the spectral data with those of related analogs. Compounds **1**–**3** were found to display activity to inhibit cell division of the fertilized eggs of the starfish *Asterina pectinifera*. 

**Table 1:** The natural products from *Astrogorgia* sp.

Structure	No.	Name	Biological Activity	Ref.
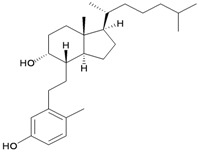	**1**	Astrogorgiadiol	Inhibited cell division of fertilized starfish (*Asterina pectinifera*) eggs at a concentration of 50 μg/mL. IC_50_ (ALK, Aurora-B, AXL, FAK, IGF1-R, MEK1 wt, MET wt, SRC, VEGF-R2) = 7.6, 25.1, 16.9, 13.2, 2.8, 48.9, 78.0, 1.9, 4.4 μM.	[2,3]
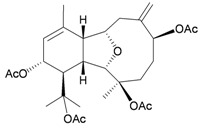	**2**	Astrogorgin	Eunicellins **2** and **3** inhibited cell division of fertilized starfish (*Asterina pectinifera*) eggs at a concentration of 10 μg/mL.	[2]
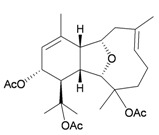	**3**	Ophirin		[2]

Furthermore, twenty-one 9,10-secosterols, including thirteen new metabolites, astrogorgols A–M (**4**–**16**), along with eight known compounds, calicoferols A–C (**17**–**19**), E (**20**), G (**21**), I (**22**), 24-exomethylenecalicoferol E (**23**), and astrogorgiadiol (**1**) and a new steroid, astrogorgol N (**24**), were isolated from the gorgonian *Astrogorgia* sp. collected from the inner coral reef in Beibuwan Bay, Guangxi province, China [3] (Table 2). The structures of new sterols **4**–**16** and **24** were determined by spectroscopic methods and by comparison of their spectral and physical data with those reported in the literature. Secosterols **1**, **9**, **17**, **18** and **23** showed inhibitory effects against a series of protein kinases. The structures of calicoferols B (**18**) [3,4], C (**19**), E (**20**) [3,5] and I (**22**) [3,6] that are shown in reference 3 should be revised as presented in the original literature. 

**Table 2:** The natural products from *Astrogorgia* sp.

Structure	No.	Name	Biological Activity	Ref.
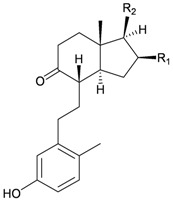	**4**	Astrogorgol A (R_1_ = H, R_2_ = SC_1_)		[3]
	**5**	Astrogorgol B (R_1_ = H, R_2_ = SC_2_)		[3]
	**6**	Astrogorgol C (R_1_ = OH, R_2_ = SC_1_)		[3]
	**7**	Astrogorgol D (R_1_ = OH, R_2_ = SC_3_)		[3]
	**17**	Calicoferol A (R_1_ = H, R_2_ = SC_3_)	IC_50_ (ALK, AXL, FAK,	[3]
			IGF1-R, MET wt, SRC,	
			VEGF-R2) = 4.2, 14.7, 9.9, 2.4,	
			47.6, 2.2, 4.6 μM.	
	**20**	Calicoferol E (R_1_ = H, R_2_ = SC_5_)		[3,5]
	**22**	Calicoferol I (R_1_ = OH, R_2_ = SC_5_)		[3,6]
	**23**	24-Exomethylenecalicoferol E	IC_50_ (ALK, AXL, FAK,	[3]
		(R_1_ = H, R_2_ = SC_4_)	IGF1-R, MET wt, SRC,	
			VEGF-R2) = 4.4, 20.2, 10.7,	
			2.3, 27.5, 1.5, 4.9 μM.	
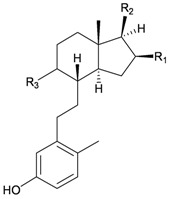	**8**	Astrogorgol E (R_1_ = H, R_2_ = SC_1_, R_3_ = β-OH)		[3]
	**9**	Astrogorgol F (R_1_ = H, R_2_ = SC_3_, R_3_ = β-OH)	IC_50_ (ALK,	[3]
			Aurora-B, AXL,	
			FAK, IGF1-R, MET	
			wt, SRC, VEGF-R2)	
			= 9.3, 38.1, 21.9,	
			16.9, 3.2, 34.0, 2.4,	
			5.0 μM	
	**10**	Astrogorgol G (R_1_ = H, R_2_ = SC_5_, R_3_ = β-OH)		[3]
	**11**	Astrogorgol H (R_1_ = H, R_2_ = SC_3_, R_3_ = α-OH)		[3]
	**12**	Astrogorgol I (R_1_ = H, R_2_ = SC_2_, R_3_ = α-OH)		[3]
	**13**	Astrogorgol J (R_1_ = H, R_2_ = SC_6_, R_3_ = α-OH)		[3]
	**14**	Astrogorgol K (R_1_ = H, R_2_ = SC_7_, R_3_ = α-OH)		[3]
	**15**	Astrogorgol L (R_1_ = OH, R_2_ = SC_3_, R_3_ = α-OH)		[3]
	**16**	Astrogorgol M (R_1_ = OH, R_2_ = SC_4_, R_3_ = α-OH)		[3]
	**18**	Calicoferol B (R_1_ = OH, R_2_ = SC_5_, R_3_ = α-OH)	IC_50_ (ALK, AXL,	[3,4]
			FAK, IGF1-R, MET	
			wt, SRC, VEGF-R2)	
			= 4.7, 32.6, 9.6, 2.5,	
			71.5, 2.2, 6.0 μM.	
	**19**	Calicoferol C (R_1_ = H, R_2_ = SC_4_, R_3_ = α-OH)		[3,5]
	**21**	Calicoferol G (R_1_ = H, R_2_ = SC8, R_3_ = α-OH)		[3]
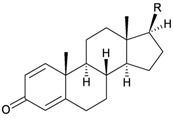	**24**	Astrogorgol N (R = SC_7_)		[3]
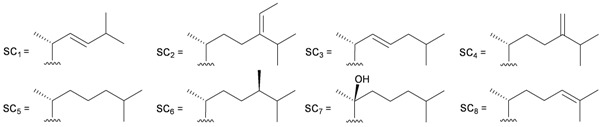				

### 2.2. Genus *Bebryce*

#### 2.2.1. *Bebryce grandicalyx*

In 1998, a new unstable sesquiterpene, bebryazulene (**25**), with a guaiane skeleton, was isolated from the gorgonian coral *B. grandicalyx*, collected at the Prevoyante Reef, Lagoon of Mayotte, Comoros Islands, Indian Ocean [7] (Table 3). The structure of guaiane **25** was assigned by spectroscopic methods. This metabolite was labile and reacted with 4-phenyl-3*H*-1,2,4-triazoline-3,5-dione to yield a triazolinedione adduct. 

**Table 3:** The natural product from *B. grandicalyx*.

Structure	No.	Name	Ref.
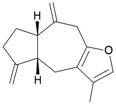	**25**	Bebryazulene	[7]

#### 2.2.2. *Bebryce indica*

*B. indica*, a gorgonian species collected off the coast of Sanya, Hainan province, China, was found to contain a new steroidal glycoside, bebrycoside (**26**) [8] (Table 4). The main structure of **26** was determined by spectral data analysis, although the stereochemistry of the C-25 chiral carbon was not determined. Bebrycoside (**26**) is the first steroidal glycoside to be isolated from the genus *Bebryce*.

**Table 4:** The natural product from *B. indica*.

Structure	No.	Name	Ref.
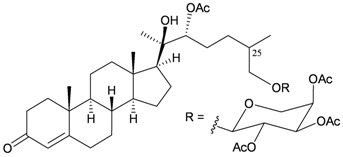	**26**	Bebrycoside	[8]

#### 2.2.3. *Bebryce* sp.

Bebryceoid A (**27**), a new trihydroxysteroid, was isolated from gorgonian *Bebryce* sp., collected off the coast of Pingtung, southern Taiwan [9] (Table 5). The structure of steroid **27** was assigned by spectroscopic methods. Bebryceoid A (**27**) exhibited weak cytotoxicity toward P388D1 and DLD-1 tumor cells. 

**Table 5:** The natural product from *Bebryce *sp.

Structure	No.	Name	Biological Activity	Ref.
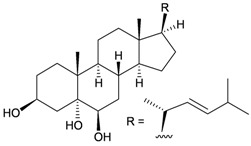	**27**	Bebryceoid A	ED_50_ (P388D1, DLD-1, CCRF-CEM, HL-60) = 18.5, 7.2, >40, >40 μg/mL	[9]

### 2.3. Genus *Echinomuricea*

#### *Echinomuricea* sp.

Two sesquiterpenoids, including new natural product (7*S*,10*R*)-(+)-10,11-epoxycurcuphenol (**28**) and known metabolite (+)-curcuphenol (**29**) [10], along with a new labdane-type diterpenoid, echinolabdane A (**30**), a new sterol, 6-*epi*-yonarasterol B (**31**) [11], a new clerodane-type diterpenoid, echinoclerodane A (**32**) [12] and a new halimane-type diterpenoid, echinohalimane A (**33**) [13], were isolated from the gorgonian coral *Echinomuricea* sp., collected off the coast of southern Taiwan (Table 6). The structures of metabolites **28**–**33** were elucidated by spectroscopic methods. Echinolabdane A (**30**) possesses a novel tetracyclic skeleton with an oxepane ring joined to an α,β-unsaturated-γ-lactone ring by a hemiketal moiety [11]. Echinolabdane A (**30**), echinoclerodane A (**32**) and echinohalimane A (**33**) are the first labdane-, clerodane- and halimane-type diterpenoids to be obtained from marine organisms belonging to the phylum Cnidaria, respectively [11,12,13]. 

**Table 6:** The natural products from *Echinomuricea *sp.

Structure	No.	Name	Biological Activity	Ref.
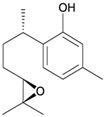	**28**	(7 *S*,10*R*)-(+)-10,11-Epoxycurcuphenol	Showed inhibitory effects on the generation of superoxide anions (inhibition rate 35.3%) and the release of elastase (inhibition rate 38.8%) at a concentration of 10 μg/mL.	[10]
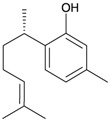	**29**	(+)-Curcuphenol	Showed inhibitory effects on the generation of superoxide anion (inhibition rate 36.9%) and the release of elastase (inhibition rate 83.6%) at a concentration of 10 μg/mL. ED_50_ (DLD-1, CCRF-CEM) = 12.5, 11.8 μg/mL.	[10]
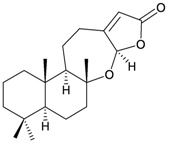	**30**	Echinolabdane A	Not active in terms of inhibition of the generation of superoxide anions (inhibition rate 2.5%) or the release of elastase (inhibition rate 1.8%) at a concentration of 10 μg/mL. IC_50_ (HL-60) = 19.1 μg/mL.	[11]
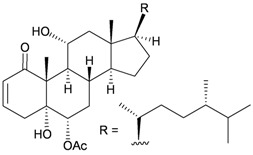	**31**	6- *epi*-Yonarasterol B	Showed significant inhibitory effects on the generation of superoxide anions (IC_50_ = 3.0 μg/mL) and the release of elastase (IC_50_ = 1.1 μg/mL).	[11]
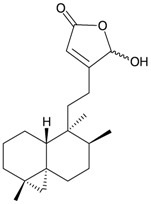	**32**	Echinoclerodane A	Showed inhibitory effects on the generation of superoxide anions (inhibition rate 68.6%) and the release of elastase (inhibition rate 35.4%) at a concentration of 10 μg/mL. IC_50_ (K562, MOLT-4, HL-60, DLD-1, LoVo, DU-145) = 37.1, 13.2, 14.9, 23.4, 21.7, 53.9 μg/mL.	[12]
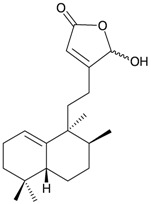	**33**	Echinohalimane A	Showed a significant inhibitory effect on the release of elastase (IC_50_ = 0.4 μg/mL).IC_50_ (K562, MOLT-4, HL-60, DLD-1, LoVo) = 6.3, 2.1, 2.1, 1.0, 0.6 μg/mL.	[13]

In biological activity experiments, sesquiterpenoid **29** displayed a significant inhibitory effect on the release of elastase by human neutrophils. This compound also exhibited weak cytotoxicity toward DLD-1 and CCRF-CEM tumor cells [9]. Steroid **31** displayed significant inhibitory effects on the generation of superoxide anions and the release of elastase by human neutrophils [11]. Clerodane **32** exhibited weak cytotoxicity toward MOLT-4 and HL-60 tumor cells and displayed a significant inhibitory effect on the generation of superoxide anions by human neutrophils [12]. Halimane **33** exhibited cytotoxicity toward K562, MOLT-4, HL-60, DLD-1 and LoVo tumor cells and displayed a significant inhibitory effect on the release of elastase by human neutrophils [13]. 

### 2.4. Genus *Euplexaura*

#### 2.4.1. *Euplexaura anastomosans*

Four new steroids of the cholestane class, anastomosacetals A–D (**34**–**37**), were obtained from the gorgonian coral *E. anastomosans*, collected off the shore of Keomun Island, South Sea Korea [14] (Table 7). The structures of steroids **34**–**37** were determined by spectroscopic methods, and these four compounds are the first examples of marine steroids possessing an unusual hemiacetal linkage formed by oxidation of the C-21 methyl group.

In addition, seven new moritoside class farnesylhydroquinone glycosides, euplexides A–G (**38**–**44**), were isolated from *E. anastomosans* [15,16] (Table 7). The structures of glycosides **38**–**44**, including their absolute stereochemistry, were elucidated by spectroscopic and chemical methods. Compounds **38**–**44** exhibited moderate cytotoxicity and antioxidant activity as well as an inhibitory effect against PLA_2_.

**Table 7:** The natural products from *E. anastomosans*.

Structure	No.	Name	Biological Activity	Ref.
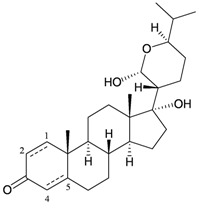	**34**	Anastomosacetal A	Steroids **34**–**37** were not toxic to P-388 cells or brine-shrimp larva.	[14]
	**35**	Anastomosacetal B (4,5-dihydro)		[14]
	**36**	Anastomosacetal C (1,2-dihydro)		[14]
	**37**	Anastomosacetal D (1,2,4,5-tetrahydro)		[14]
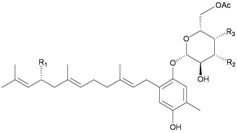	**38**	Euplexide A (R_1_ = OH, R_2_ = R_3_ = OAc)	Glycosides **38**–**40**, **43**, **44** exhibited cytotoxicity toward K462 cells (IC_50_ = 2.6, 3.1, 5.2, 8.7, 11.3 μg/mL). Glycosides **38**–**40** displayed antioxidant activity of 3.4, 3.6 and 3.5 times, respectively, that of superoxide dismutase (SOD) at a concentration of 10 μg/300 μL. Glycosides **38**, **39**, **43**, **44** exhibited 52, 71, 47 and 58%, respectively, inhibition of PLA_2_ at a concentration of 50 μg/mL.	[15]		
	**39**	Euplexide B (R_1_ = R_2_ = R_3_ = OAc)		[15]
	**40**	Euplexide C (R_1_ = H, R_2_ = R_3_ = OAc)		[15]
	**43**	Euplexide F (R_1_ = H, R_2_ = OH, R_3_ = OAc)		[16]
	**44**	Euplexide G (R_1_ = H, R_2_ = OAc, R_3_ = OH)		[16]
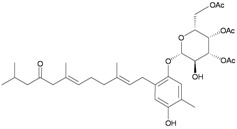	**41**	Euplexide D	IC_50_ (K462) = 8.1 μg/mL.	[15]	
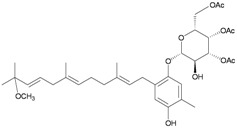	**42**	Euplexide E	IC_50_ (K462) = 9.4 μg/mL. Displayed antioxidant activity of 3.1 times that of superoxide dismutase (SOD) at a concentration of 10 μg/300 μL.	[15]

#### 2.4.2. *Euplexaura erecta*

A prostaglandin derivative, PGF_2α_ (**45**), was isolated from the gorgonian coral *E. erecta* collected at Shimoda, Sagami Bay, Japan [17] (Table 8), and this compound was proven to be the active component in *E. erecta*. This finding is the first demonstration that gorgonian corals containing prostaglandins are not limited to species in the Caribbean area.

Furthermore, a bluish-violet oil, guaiazulene (**46**), was isolated from *E. erecta* collected at Enoshima Island, Kanagawa, Japan [18] (Table 8). The structure of guaiazulene (**46**) from *E. erecta* was determined by spectroscopic methods and by comparison of the spectral data with those of reported data. This is the first isolation of guaiazulene from an animal, and this compound showed mild antimicrobial activity [18].

**Table 8:** The natural products from *E. erecta*.

Structure	No.	Name	Biological Activity	Ref.
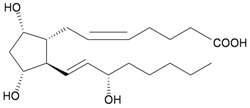	**45**	PGF_2α_	Contracting activity towards isolated guinea-pig ileum strips.	[17]
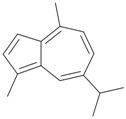	**46**	Guaiazulene	Showed mild activity against fungi, gram-positive and gram-negative bacteria.	[18]

#### 2.4.3. *Euplexaura flava*

Four new unnamed fatty acid derivatives **47**–**50**, which contain a butenolide moiety, were isolated from the gorgonian coral *E. flava*, collected at the coral reef of Ishigaki Island, Okinawa, Japan. The structures of butenolides **47**–**50** were elucidated by spectroscopic and chemical methods [19] (Table 9).

**Table 9:** The natural products from *E. flava*.

Structure	No.	Name	Ref.
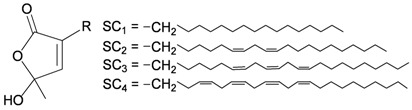	**47**	R = SC_1_	[19]
	**48**	R = SC_2_	[19]
	**49**	R = SC_3_	[19]
	**50**	R = SC_4_	[19]

#### 2.4.4. *Euplexaura nuttingi*

Six new tetraprenylated purine alkaloids, nuttingins A–F (**51**–**56**), were isolated together with five new compounds, malonganenones D–H (**57**–**61**), and three known metabolites, malonganenones A–C (**62**–**64**), from the gorgonian coral *E. nuttingi* collected in Uvinage, Pemba Island, Tanzania. The structures of compounds **51**–**64** were elucidated by interpretation of spectral data [20] (Table 10). Mixtures of nuttingins A and B (**51** and **52**), C–E (**53**–**55**), malonganenones D and E (**57** and **58**), and F and G (**59** and **60**) have been found to inhibit growth of K562 and UT7 tumor cells. Nuttingins A–E (**51**–**55**) and malonganenones D–H (**57**–**61**) induce apoptosis in transformed mammalian cells [20]. 

**Table 10:** The natural products from *E. nuttingi*.

Structure	No.	Name	Biological Activity	Ref.
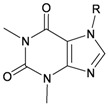	**51**	Nuttingin A (R = SC_1_)	Compounds **51**–**55** and **57**–**61** induce apoptosis in transformed mammalian cells at a concentration of 1.25 μg/mL. Mixtures of compounds **51** and **52** displayed inhibitory activity on the proliferation of UT7 and K562 cell lines, although they were approximately 3-fold less potent than mixtures of compounds **53**–**55**.	[20]
	**52**	Nuttingin B (R = SC_3_)		[20]
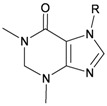	**53**	Nuttingin C (R = SC_1_)	Mixtures of compounds **53**–**55** induced 50% inhibition of cell growth in UT7 cells and 30% in K562 cells after 48 h of exposure at a concentration of 0.4 μg/mL.	[20]
	**54**	Nuttingin D (R = SC_2_)		[20]
	**55**	Nuttingin E (R = SC_3_)		[20]
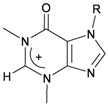	**56**	Nuttingin F (R = SC_2_)		[20]
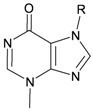	**57**	Malonganenone D (R = SC_1_)	Mixtures of compounds **57** and **58** displayed inhibitory activity on the proliferation of UT7 and K562 cell lines, although they were approximately 3-fold less potent than mixtures of compounds **53**–**55**.	[20]
	**58**	Malonganenone E (R = SC_2_)		[20]
	**62**	Malonganenone A (R = SC_3_)		[20]
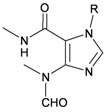	**59**	Malonganenone F (R = SC_1_)	Mixtures of compounds **59** and **60** displayed inhibitory activity on the proliferation of UT7 and K562 cell lines, although they were approximately 3-fold less potent than mixtures of compounds **53**–**55**.	[20]
	**60**	Malonganenone G (R = SC_2_)		[20]
	**63**	Malonganenone B (R = SC_3_)		[20]
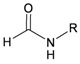	**61**	Malonganenone H (R = SC_2_)		[20]
	**64**	Malonganenone C (R = SC_3_)		[20]
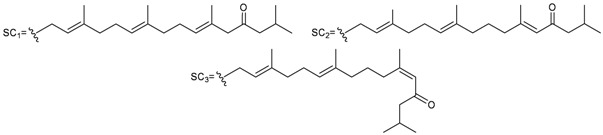				

#### 2.4.5. *Euplexaura* sp.

Moritoside (**65**), a new hydroquinone glycoside derivative was isolated from the gorgonian *Euplexaura* sp., collected near Morito beach in the Gulf of Sagami, Japan. The structure of glycoside **65** was determined by spectroscopic and chemical methods [21] (Table 11). This is the first example of the occurrence of D-altrose in natural products, and this compound inhibits the first cell division of fertilized starfish (*Asterina pectinifera*) eggs.

**Table 11:** The natural product from *Euplexaura *sp.

Structure	No.	Name	Biological Activity	Ref.
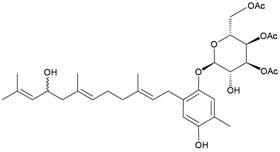	**65**	Moritoside	Inhibits the first cell division of fertilized starfish (*Asterina pectinifera*) eggs at a concentration of 1 μg/mL.	[21]

### 2.5. Genus *Menella*

#### 2.5.1. *Menella spinifera*

The gorgonian *M. spinifera* collected off the South China Sea was found to contain six known compounds, including batyl alcohol (**66**) [22,23], picolinic acid *N*-methyl betaine (**67**) [23,24], *n*-hexadecanol (**68**) [23], 3β-hydroxy-5α-pregnane-20-one (**69**) [23], 9*H*-purin-6-amino-*N*-9-dimethyl (**70**) [23] and thymidine (**71**) [23] (Table 12). The structures of compounds **66**–**71** were elucidated by spectroscopic methods.

**Table 12:** The natural products from *M. spinifera*.

Structure	No.	Name	Ref.
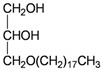	**66**	Batyl alcohol	[22,23]
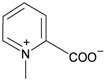	**67**	Picolinic acid *N*-methyl betaine	[23,24]
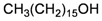	**68**	*n*-Hexadecanol	[23]
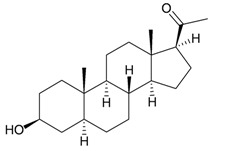	**69**	3β-Hydroxy-5α-pregnane-20-one	[23]
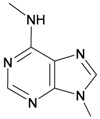	**70**	9 *H*-Purin-6-amino-*N*-9-dimethyl	[23]
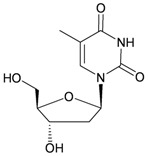	**71**	Thymidine	[23]

#### 2.5.2. *Menella verrucosa*

Four new highly-oxygenated guaiane lactones, menverins A–D (**72**–**75**) [25], and two new polyoxygenated steroids, menellsteroids A (**76**) and B (**77**) [26] (Table 13), were isolated from the gorgonian *M. verrucosa*, collected along the coast of Xiaodong Hai, Hainan province, China. The structures of metabolites **72**–**77** were established by spectroscopic methods. In a later study, menellsteroid A (**76**) was found to exhibit modest anti-inflammatory inhibition of lipopolysaccharide (LPS)-induced nitric oxide (NO) production in RAW264.7 macrophages [27].

**Table 13:** The natural products from *M. verrucosa*.

Structure	No.	Name	Biological Activity	Ref.
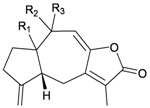	**72**	Menverin A (R_1_ = α-H, R_2_ = β-OH, R_3_ = α-methyl)		[25]
	**73**	Menverin B (R_1_ = α-H, R_2_ = β-methyl, R_3_ = α-OH)		[25]
	**74**	Menverin C (R_1_ = α-OH, R_2_ = β-OH, R_3_ = α-methyl)		[25]
	**75**	Menverin D (R_1_ = R_2_ = β-OH, R_3_ = α-methyl)		[25]
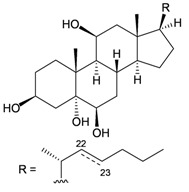	**76**	Menellsteroid A (22,23-dihydro)	Exhibited a modest inhibitory effect with an IC_50_ of 33.9 μM compared to the positive control aminoguanidine, with an IC_50_ = 25.0 μM.	[26,27]
	**77**	Menellsteroid B		[26]

#### 2.5.3. *Menella* sp.

Li *et al.*, isolated four new highly-oxygenated guaiane lactones, 1-epimenverin B (**78**), menverin F (**79**), 1-deoxymenverin F (**80**) and menverin G (**81**), along with two known guaiane analogs, menverins B (**73**) and C (**74**), from the gorgonian *Menella* sp., collected off the Lingshui Bay, Hainan province, China [28] (Table 14). The structures of new guaianes **78**–**81** were elucidated by spectroscopic methods and by comparison with those of known analogs. 

**Table 14:** The natural products from *Menella *sp.

Structure	No.	Name	Ref.
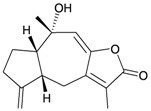	**78**	1-Epimenverin B	[28]
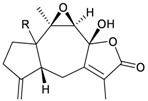	**79**	Menverin F (R = α-OH)	[28]
	**80**	1-Deoxymenverin F (R = α-H)	[28]
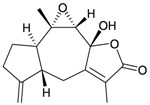	**81**	Menverin G	[28]

A chemical investigation of the gorgonian *Menella* sp., collected off Meishan Island, Hainan province, China, resulted in a novel highly-oxygenated racemate with a C8 skeleton, menellin A (**82**), a new tetrahydroxysteroid, menellsteroid C (**83**), a new natural product, 1β,3β,5α-trihydroxy-cholestan-6-one (**84**) and seven known compounds, menellsteroid A (**76**), cholestan-3β,5α,6β-triol (**85**), cholestan-1β,3β,5α,6β-tetrol (**86**), nephalsterol (**87**), cholestan-3β-5-en-6-one (**88**) and junceellolides B (**89**) and D (**90**) [27] (Table 15). The structures of the above compounds were elucidated by spectroscopic methods and by comparison of the spectral data with those of known analogs. The structure, including the relative stereochemistry, of menellin A (**82**) was further confirmed by single-crystal X-ray diffraction analysis. As already reported for menellsteroid A (**76**), menellin A (**82**) exhibited modest anti-inflammatory inhibition of lipopolysaccharide (LPS)-induced nitric oxide (NO) production in RAW264.7 macrophages.

Seven pregnane steroids, 3α-hydroxy-5β-pregnan-20-one (**91**), 3β-hydroxy-5α-pregnan-20-one (**92**), 3β-hydroxy-pregnan-5-en-20-one (**93**), 5β-pregnan-3,20-dione (**94**), 5α-pregnan-3,20-dione (**95**), pregnan-4-en-3,20-dione (**96**) and pregnan-1,4-dien-3,20-one (**97**), were isolated from the gorgonian *Menella* sp., collected off Meishan Island, Sanya Bay, Hainan province, China [29] (Table 16). The structures of steroids **91**–**97** were elucidated by spectroscopic methods and by comparison with those of known analogs. The NMR data of steroid **97** are reported for the first time in this study. 

**Table 15:** The natural products from *Menella *sp.

Structure	No.	Name	Biological Activity		Ref.	
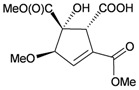	**82**	Menellin A	Exhibited a modest inhibitory effect (IC_50_ = 71.3 μM) compared to the positive control aminoguanidine (IC_50_ = 25.0 μM). There were no obvious scavenging effects for compounds **76** and **82**–**90** on the antioxidant capacity in a radical DPPH free-radical assay.		[27]	
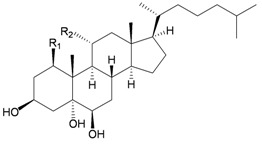	**83**	Menellsteroid C (R_1_ = H, R_2_ = OH)			[27]	
	**85**	Cholestan-3β,5α,6β-triol (R_1_ = R_2_ = H)			[27]	
	**86**	Cholestan-1β,3β,5α,6β-tetrol (R_1_ = OH, R_2_ = H)			[27]	
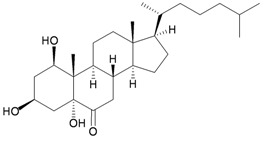	**84**	Menellsteroid D (1β,3β,5α-trihydroxycholestan-6-one)			[27]	
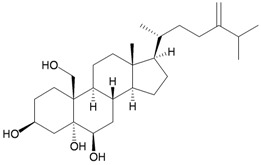	**87**	Nephalsterol			[27]	
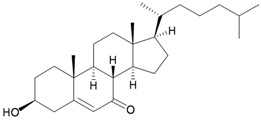	**88**	Cholestan-3β-5-en-6-one			[27]	
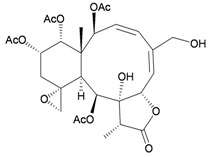	**89**	Junceellolide B			[27]	
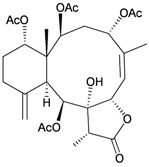	**90**	Junceellolide D			[27]

**Table 16:** The natural products from *Menella *sp.

Structure	No.	Name	Ref.
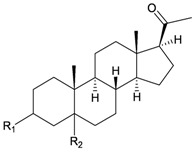	**91**	3α-Hydroxy-5β-pregnan-20-one (R_1_ = α-OH, R_2_ = β-H)	[29]
	**92**	3β-Hydroxy-5α-pregnan-20-one (R_1_ = β-OH, R_2_ = α-H)	[29]
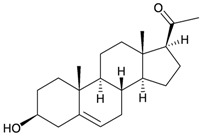	**93**	3β-Hydroxy-pregnan-5-en-20-one	[29]
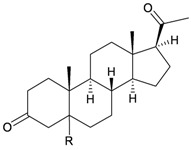	**94**	5β-Pregnan-3,20-dione (R = β-H)	[29]
	**95**	5α-Pregnan-3,20-dione (R = α-H)	[29]
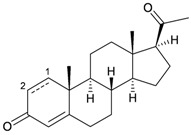	**96**	Pregnan-4-en-3,20-dione (1,2-dihydro)	[29]
	**97**	Pregnan-1,4-dien-3,20-dione	[29]

Eight sesquiterpenoids, including seven new compounds, (–)-hydroxylindestrenolide (**98**) [30], menelloides A–E (**99**–**103**) [31,32,33] and (+)-chloranthalactone B (**104**) [31], along with a known metabolite, seco-germacrane anhydride (**105**) [34] (Table 17), were isolated from the Formosan gorgonian *Menella* sp., collected by trawling off the coast of southern Taiwan. (–)-Hydroxy-lindestrenolide (**98**) and (+)-chloranthalactone B (**104**) were proven to be enantiomers of the known sesquiterpenoids (+)-hydroxylindestrenolide and chloranthalactone B, respectively [30,31]. Menelloide A (**99**) was found to possess a new carbon skeleton [31]. Seco-germacrane anhydride (**105**) was a known metabolite and there have been no reports of seco-germacrane anhydride (**105**) being obtained from any marine organism previously [34]. Several of these compounds displayed inhibitory effects on the generation of superoxide anions and the release of elastase by human neutrophils.

**Table 17:** The natural products from *Menella *sp.

Structure	No.	Name	Biological Activity	Ref.
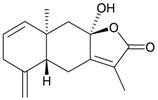	**98**	(–)-Hydroxylindestrenolide	Displayed a weak inhibitory effect on the generation of superoxide anions (inhibition rate 13.4%) at a concentration of 10 μg/mL.	[30]
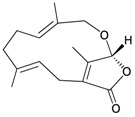	**99**	Menelloide A	Displayed a weak inhibitory effect on the generation of superoxide anions (27.6%) at a concentration of 10 μg/mL.	[31]
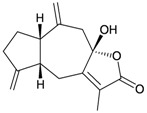	**100**	Menelloide B	Not active in terms of inhibition of the generation of superoxide anions (inhibition rate 2.9%) and the release of elastase (inhibition rate 0.7%) at a concentration of 10 μg/mL.	[31]
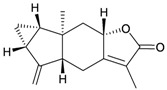	**101**	Menelloide C		[32]
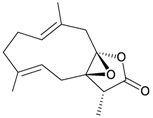	**102**	Menelloide D	Displayed a weak inhibitory effect on the release of elastase (inhibition rate 10.5%) at a concentration of 10 μg/mL.	[32]
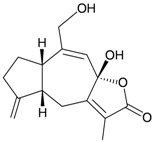	**103**	Menelloide E	Displayed weak inhibitory effects on the generation of superoxide anions (inhibition rate 19.9%) and the release of elastase (inhibition rate 27.0%) at a concentration of 10 μg/mL.	[33]
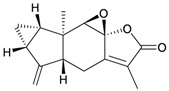	**104**	(+)-Chloranthalactone B	Displayed a weak inhibitory effect on the generation of superoxide anions (inhibition rate 16.5%), but was not active in terms of inhibition of the release of elastase (inhibition rate 6.6%) at a concentration of 10 μg/mL.	[31]
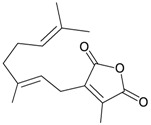	**105**	Seco-germacrane anhydride		[34]

## 3. Conclusions

The search for bioactive natural products from marine organisms has been remarkably successful, and octocorals have been proven to be rich sources of natural products with potential biomedical application [35,36,37]. In particular, the data reported in this review indicate that terpenoid and steroid derivatives represent the major chemical classes occurring in Indo-Pacific octocoral species belonging to the family Plexauridae. Among the 105 isolated metabolites, in fact, 49 compounds are terpenoid analogs (46.7%) and 45 compounds are steroid metabolites (42.9%). These compounds continue to attract attention owing to their structural novelty, complexity and interesting bioactivities. 
